# An interpretable machine learning framework using multi-phase computed tomography for differentiation of adrenal lipid-poor adenomas and pheochromocytomas

**DOI:** 10.3389/frai.2026.1915569

**Published:** 2026-09-08

**Authors:** Panliang Zhao, Fangmei Zhu, Cheng Yan, Zongfeng Niu, Linyang He, Zongyu Xie, Jian Wang

**Affiliations:** 1 Department of Radiology, Tongde Hospital of Zhejiang Province Affiliated to Zhejiang Chinese Medical University, Hangzhou, Zhejiang, China; 2 Department of Radiology, Affiliated Hangzhou First People's Hospital, Westlake University School of Medicine, Hangzhou, Zhejiang, China; 3 Department of Radiology, Sir Run Run Shaw Hospital, Zhejiang University School of Medicine, Hangzhou, Zhejiang, China; 4 Jianpei Technology, Hangzhou, Zhejiang, China; 5 Department of Radiology, The First Affiliated Hospital of Bengbu Medical University, Bengbu, Anhui, China

**Keywords:** adrenal gland, artificial intelligence, computed tomography, lipid-poor adenoma, machine learning, pheochromocytoma

## Abstract

**Objective:**

To develop and validate an interpretable machine learning (ML) framework that differentiates adrenal lipid-poor adenomas (LPAs) from pheochromocytomas (PHEOs) using radiomic features derived from multi-phase contrast-enhanced computed tomography (CECT).

**Methods:**

This retrospective study included 229 patients with pathologically confirmed adrenal tumours (132 LPAs, 97 PHEOs). Lesions were stratified into washout (absolute percentage washout [APW] > 60%) and non-washout (APW ≤ 60%) cohorts according to established criteria. We trained decision tree (DT), support vector machine (SVM), and logistic regression (LR) models using a predefined set of clinically relevant CT features. The inherently interpretable DT model was further dissected to revealr key discriminative features and its underlying decision logic. Model performance was evaluated using the area under the curve (AUC) with 95% confidence intervals.

**Results:**

The DT model achieved excellent diagnostic performance, with an AUC of 0.977 (95% CI: 0.934–1.000) in the washout group and 0.983 (95% CI: 0.962–1.000) in the non-washout group. Feature importance analysis identified cystic degeneration as the most influential discriminator, followed by enhancement potential (EP) and baseline attenuation (CTu). The DT’s hierarchical decision rules provided clear and clinically transparent pathways.

**Conclusion:**

This interpretable ML framework accurately distinguishes LPAs from PHEOs. The DT model strikes an optimal balance between high diagnostic accuracy and inherent interpretability, offering a practical tool that may enhance clinical confidence when managing indeterminate adrenal lesions.

## Introduction

The widespread use of cross-sectional imaging has substantially increased the detection rate of adrenal incidentalomas (AIs), with autopsy studies reporting a prevalence of up to 8.7% (Kloos et al., 1995). The majority of these incidentally discovered lesions are nonfunctional adenomas (80%); other pathologies include subclinical Cushing’s syndrome (5.0%), pheochromocytomas (PHEOs, 5.0%), aldosterone-producing adenomas (1%), adrenal cortical carcinomas (<5.0%), and metastases (2.5%) (Young, 2000; Grumbach et al., 2003).

Current clinical guidelines recommend that adrenal incidentalomas with an unenhanced computed tomography (CT) attenuation of ≤10 Hounsfield Units (HU) typically do not warrant further imaging evaluation (Fassnacht et al., 2023; Rowe et al., 2023; Canu et al., 2019; Zeiger et al., 2009; Buitenwerf et al., 2018; Gruber et al., 2020). Notably, almost all PHEOs exhibit attenuation values well above 10 HU on unenhanced CT and remain hyperdense on contrast-enhanced images (Canu et al., 2019; Buitenwerf et al., 2018; Gruber et al., 2020). Consequently, while most nonfunctional adenomas can be managed conservatively, PHEOs often require surgical or other interventions owing to their malignant potential and risk of recurrence or metastasis (Lider Burciulescu et al., 2024; Schlegel et al., 2024). This stark divergence in management strategies between lipid-poor adenomas (LPAs, attenuation >10 HU) and PHEOs underscores the critical need for accurate non-invasive differentiation.

Recent studies have confirmed that a considerable proportion of PHEOs exhibit washout characteristics that overlap with those of adenomas, thereby complicating traditional CT-based differentiation (Shan et al., 2025). Moreover, atypical adrenal masses with lipid-poor features carry a non-negligible malignant potential, underscoring the need for more precise diagnostic tools (Balderrama-Brondani et al., 2025).

Machine learning (ML) has recently emerged as a powerful tool for deciphering complex patterns within medical images, offering substantial gains in diagnostic accuracy. Previous studies have applied ML algorithms to adrenal tumour characterisation, primarily using features derived from non-contrast or single-phase CT (Liu et al., 2022; Xiao et al., 2023). For instance, a CT radiomics analysis by Mendi and Gülbay, has demonstrated that models based solely on non-contrast or single-phase contrast-enhanced CT (CECT) can achieve commendable diagnostic performance (e.g., random forest area under the curve [AUC] 0.975) (Mendi and Gulbay, 2023), highlighting the potential of simplified imaging protocols to reduce radiation and contrast exposure (Glazer et al., 2025). However, by design, these approaches fail to harness the dynamic enhancement information captured by multi-phase protocols. Consequently, the specific diagnostic utility of washout characteristics—such as absolute percentage washout (APW)—for adrenal lesion subtyping remains underexplored and contentious (Woo et al., 2018).

It is well recognised that the diagnostic workup for AIs—particularly for excluding PHEO—relies heavily on biochemical testing for catecholamines or metanephrines (Fassnacht et al., 2023; Gruber et al., 2020). However, our objective is not to supplant this essential biochemical profiling, but rather to explore the standalone diagnostic potential of multi-phase CECT features. A robust radiological model is particularly valuable in scenarios where biochemical tests are pending, inconclusive, or when clinical suspicion for a secretory tumour remains low despite indeterminate imaging findings. By offering an objective assessment based on readily available CT data, such a model could serve as a useful triage tool, prompting more urgent and targeted biochemical evaluation when warranted.

To address these gaps, we developed an interpretable ML framework that harnesses a comprehensive set of quantitative and qualitative features extracted from multi-phase CECT. Our primary contribution lies not merely in the use of multi-phase CT, but in employing an interpretable decision tree (DT) model to distil this multiparametric data into concise, clinically logical decision pathways. Furthermore, we demonstrate how the key discriminative features shift distinctly between washout and non-washout lesions, offering novel insights into their underlying pathophysiology. This study aims not only to achieve high diagnostic accuracy but also to provide transparent insights into the model’s decision-making process, with the goal of building clinician trust and facilitating clinical adoption.

## Methods

### Patient selection

**Figure 1:**
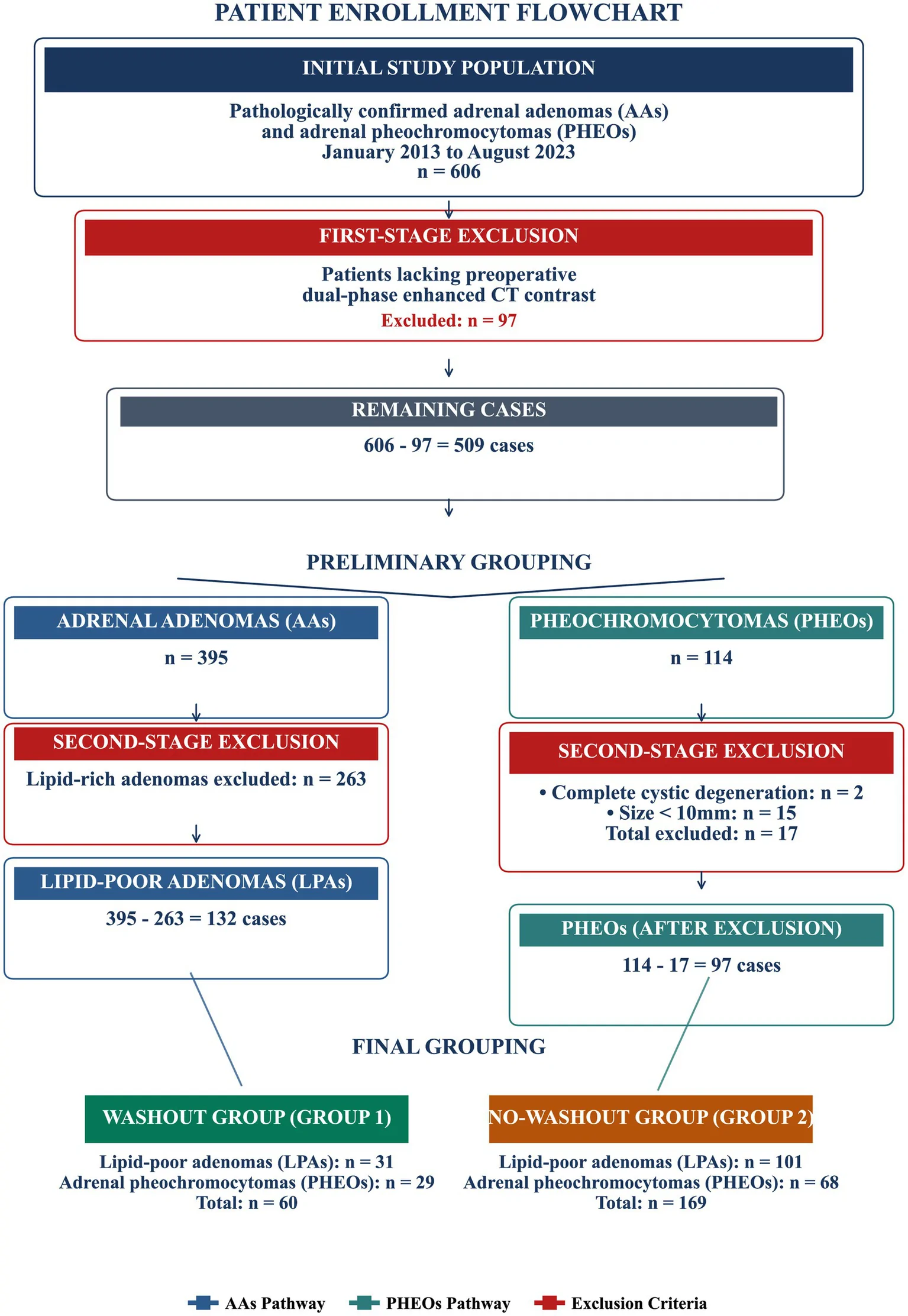
Patient selection flowchart.

This retrospective study was conducted in accordance with the Declaration of Helsinki (as revised in 2013) and received approval from the Institutional Ethics Committee of Tongde Hospital, Zhejiang Province (Approval No. 2022183-JY). The committee granted a waiver of informed consent owing to the retrospective use of anonymised data. We identified consecutive patients from our institutional pathology database who had histopathologically confirmed adrenal tumours—either LPAs or PHEOs—and were enrolled between January 2013 and August 2023. Exclusion criteria comprised substandard CT image quality, incomplete clinical data, or the presence of concurrent adrenal pathologies such as metastases. The final cohort comprised 229 patients (132 LPAs, 97 PHEOs). Based on an established APW threshold of 60% (Shan et al., 2025), lesions were stratified into a washout group (APW > 60%; *n* = 60; 31 LPAs and 29 PHEOs) and a non-washout group (APW ≤ 60%; *n* = 169; 101 LPAs and 68 PHEOs). The patient selection process is summarized in Figure 1.

### CT image acquisition

Multiphase CT examinations were performed using multidetector scanners from Siemens Healthineers (SOMATOM Sensation 16 and SOMATOM Definition Flash) and GE Healthcare (LightSpeed VCT), with Siemens and GE scanners used in an approximate 3:2 ratio. Following the establishment of intravenous access, a weight-adjusted bolus (0.8–1.2 mL/kg) of the non-ionic contrast agent Iohexol (350 mg I/mL) was administered at 2.5–3.0 mL/s. Arterial phase imaging was triggered by an automatic bolus-tracking technique, with a region of interest placed in the descending aorta, and a trigger threshold of 100 HU. This was followed by sequential acquisitions in the arterial (25–40 s) and portal venous (55–60 s) phases. The field of view was tailored to patient anatomy, typically ranging from 250 to 350 mm. All scans were acquired at 120 kV with automatic tube current modulation (200–300 mA). Initial acquisitions used a 5-mm slice collimation. For subsequent analysis, all raw data were reconstructed to a uniform 1.5-mm slice thickness to ensure dimensional consistency across the cohort.

### Lesion stratification and washout analysis

Lesions were stratified using the APW, calculated from multiphase CT data using the standard formula:

APW=[(CTa—CTv)/(CTa—CTu)]×100%

Where CTu, CTa, and CTv represent the CT attenuation values (in HU) in the unenhanced, arterial, and venous phases, respectively. For a comprehensive assessment, the relative percentage washout (RPW) was also computed as follows (Shan et al., 2025).

RPW=[(CTa—CTv)/CTa]×100%.

The decision to stratify lesions into washout and non-washout cohorts was based on the established clinical APW threshold of 60%. Given that a substantial proportion of PHEOs exhibit washout characteristics overlapping with adenomas (Shan et al., 2025; Woo et al., 2018; Patel et al., 2013), we sought to explore whether the diagnostic contribution of other imaging biomarkers differs according to the tumour’s washout phenotype—a hypothesis that requires subgroup-specific modelling.

### Image analysis and feature extraction

Two board-certified abdominal radiologists (with 12 and 15 years of experience), blinded to the pathological diagnoses, independently evaluated all CT images on a standardised post-processing workstation (Syngo.via, Siemens Healthineers).

All measurements were performed on a single representative axial slice showing the largest tumour cross-section. The radiologists placed a circular region of interest (ROI) larger than 0.05 cm^2^ within the lesion to obtain mean CT attenuation values (CTu, CTa, CTv), carefully sampling the solid parenchyma while avoiding necrotic areas, calcifications, haemorrhage, and the lesion periphery. In larger lesions, multiple ROIs or a single enlarged ROI were used to ensure adequate sampling. Each reader performed two measurement cycles in a single session; the average of these two cycles was calculated, and the final value for each parameter was derived from the mean of the two readers’ averages.

Inter-observer agreement was excellent for continuous variables (e.g., CTu, CTa, CTv), with an intraclass correlation coefficient (ICC) (2,1) of 0.89 (95% confidence interval [CI]: 0.85–0.92). Agreement for categorical variables (e.g., cystic degeneration) was substantial, with a Cohen’s kappa (κ) of 0.76.

The evaluated CT parameters included:

Morphological characteristics: Long diameter (LD), short diameter (SD), LD/SD ratio (categorized as Round: ≤1.2; Oval: >1.2; Irregular contour), and laterality.

Enhancement patterns: CTu, CTa, CTv, APW, RPW, degree of enhancement in the arterial phase (DEAP = CTa—CTu), and degree of enhancement in the portal venous phase (DEVP = CTv—CTu).

Specialized diagnostic criteria: Cystic degeneration was defined as areas exhibiting CTu < 20 HU, DEAP < 10 HU, and DEVP < 10 HU (Boland et al., 1998). Enhancement potential (EP) was defined as EP = max(DEAP, DEVP), representing the peak absolute enhancement across the arterial and portal venous phases.

## The ML workflow

### Feature set and model development

We developed DT, SVM, and LR models using a predefined set of CT features, all selected for their established clinical relevance in adrenal lesion characterisation (Liu et al., 2022; Mendi and Gulbay, 2023; Boland et al., 1998). This set comprised:

Quantitative attenuation values: CTu, CTa, CTv.

Derived enhancement parameters: APW, RPW, DEAP, DEVP, EP.

Basic morphological metrics: LD and SD.

Key qualitative features: The presence of cystic degeneration and intratumoural vessels.

This feature set was used to train three distinct classifier models: DT, SVM, and LR. Given its inherent interpretability, the DT model was subjected to further analysis to identify the most discriminative features and decision thresholds through its feature importance scores and rule structure.

Model development and evaluation employed stratified 5-fold cross-validation with a 70/30 split for training and testing in each fold. Hyperparameters were optimised via a grid search. The DT model used the Gini impurity criterion, with max_depth set to 3 for the washout group and 5 for the non-washout group. The SVM used a Radial Basis Function kernel, with the cost parameter C set to 1.0 and 10.0 for the washout and non-washout groups, respectively. The LR model used an L2 penalty, with regularization strength set to 0.01 (washout) and 0.1 (non-washout). To mitigate the risk of overfitting, we adopted a parsimonious feature selection strategy, limiting our model inputs to a predefined set of clinically established CT parameters (approximately 10), combined with stratified 5-fold cross-validation. The entire ML workflow was implemented in Python 3.12 using the scikit-learn library (version 1.3.0).

### Statistical analysis

Statistical analyses were conducted using IBM SPSS Statistics (Version 26.0) and Python 3.12. Continuous variables were tested for normality with the Shapiro–Wilk test. Normally distributed data are presented as the mean ± standard deviation and compared using the Student’s *t*-test; non-normally distributed data are expressed as the median (interquartile range) and compared with the Mann–Whitney *U* test. Categorical variables are reported as frequencies and compared using the Chi-square or Fisher’s exact test, as appropriate.

Model performance was evaluated using sensitivity, specificity, accuracy, and the AUC, the latter reported with its 95% CI. Pairwise comparisons of AUC values between models were performed using the DeLong test. A *p*-value less than 0.05 was considered statistically significant.

## Results

### Patient characteristics and model performance

**Figure 2:**
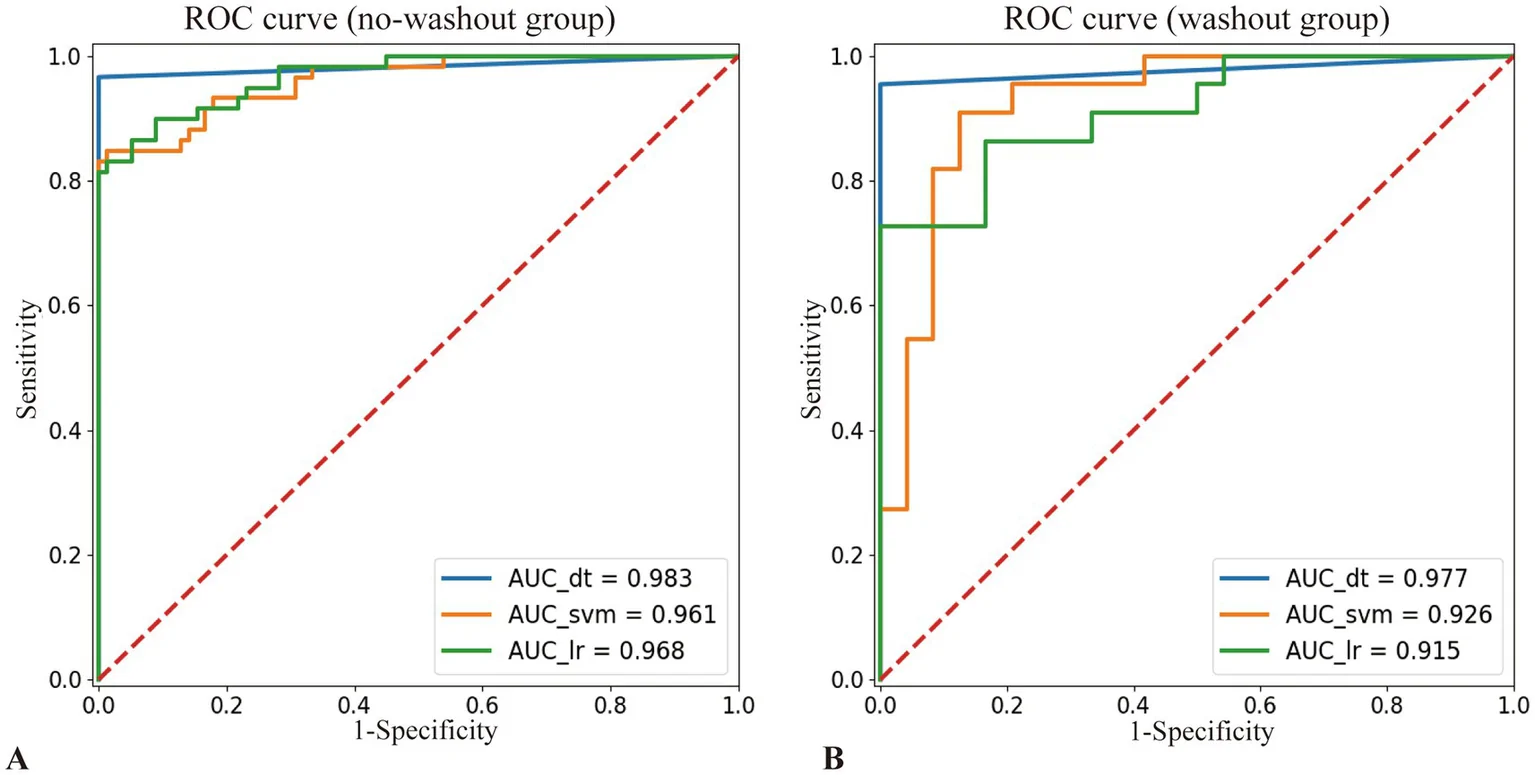
ROC curves comparing model performance in the **(A)** non-washout and **(B)** washout cohorts.

The baseline characteristics of the patients in the washout and non-washout cohorts are summarized in Tables 1, 2, respectively. All three ML models demonstrated high diagnostic performance in differentiating LPAs from PHEOs (Table 3, Figure 2).

**Table 1:** Demographic characteristics and quantitative CT features between LPAs and PHEOs.

Characteristics	Washout group		*p*-value	Non-washout group		*p*-value	ALL subjects group		*p*-value
	LPAs (*n* ﹦31)	PHEOs (*n* ﹦29)		LPAs (*n* ﹦101)	PHEOs (*n* ﹦68)		LPAs (*n* ﹦132)	PHEOs (*n* ﹦97)	
Age	54.23 ± 9.21	46.28 ± 13.8	0.012*	50.94 ± 11.77	50.56 ± 13.940	0.848*	51.71 ± 11.273	49.27 ± 13.96	0.16*
Attenuation									
CTu	26.90 (19.90, 35.80)	38.80 (32.70,43.10)	**<0.001†**	28.98 ± 10.02	38.41 ± 7.35	<0.001*	28.98 ± 10.02	38.41 ± 7.35	<0.001*
CTa	88.40 (68.35,105.00)	127.00 (105.80,165.1)	**<0.001†**	89.19 ± 25.64	136.21 ± 41.16	<0.001*	89.19 ± 25.64	136.21 ± 41.16	<0.001*
CTv	78.05 ± 23.72	110.86 ± 31.43	<0.001*	81.00 (68.10,93.70)	90.85 (73.20,112.20)	**0.002†**	80.45 (66.55,93.65)	98.00 (77.70,120.60)	**<0.001†**
Degree of enhancement									
DEAP	60.21 ± 19.63	97.80 ± 41.36	<0.001*	31.30 (22.90,45.30)	31.55 (18.60,50.60)	0.965**†**	37.15 (25.30,51.90)	44.50 (25.10,81.70)	**0.040†**
DEVP	78.05 ± 23.72	110.86 ± 31.43	0.003*	55.20 (47.80,67.10)	49.10 (33.45,76.50)	0.152**†**	53.70 (44.85,65.30)	53.90 (37.90,82.50)	0.675**†**
DEmax	59.70 (47.35,71.10)	83.80 (61.20,129.90)	**<0.001†**	55.20 (47.80,67.10)	49.10 (34.10,76.50)	0.162**†**	55.65 (47.75,69.80)	61.20 (39.90,89.20)	0.269**†**
EP	2.10 (1.72,2.58)	2.44 (1.64,3.67)	0.355†	2.51 (1.75,3.43)	1.33 (0.84,2.11)	**<0.001†**	2.31 (1.75,3.22)	1.63 (1.00,2.68)	**<0.001†**
APW	16.05 (8.46, 23.09)	24.53 (8.64,37.97)	0.264**†**	−73.08 (−140.59,−26.75)	−54.93 (−116.11,−23.46)	0.245**†**	−42.51 (−6112.94,−3.46)	−24.42 (−84.90,6.22)	0.076†
RPW	10.81 (5.70,16.00)	17.36 (5.56,24.78)	0.201**†**	−42.23 (−71.05,−18.15)	−22.55 (−36.80,−11.05)	**<0.001†**	−25.81 (−60.31,−2.19)	−13.06 (−30.21,3.44)	**0.001†**
Size									
LD	19.00 (14.50,24.50)	34.00 (23.00,47.00)	**0.002†**	23.00 (18.00,27.00)	38.50 (29.50,53.00)	**<0.001 †**	22.00 (17.00,27.00)	36.00 (25.00,50.00)	**<0.001†**
SD	15.00 (11.50,19.00)	25.00 (14.00,36.00)	**0.001†**	18.00 (14.00,22.00)	32.00 (23.00,45.00)	**<0.001†**	17.50 (13.00,22.00)	30.00 (23.00,41.00)	**<0.001†**
LD/SD	1.25 (1.12, 1.39)	1.17 (1.13,1.35)	0.706†	1.21 (1.09,1.35)	1.17 (1.08,1.28)	0.196†	1.23 (1.10,1.37)	1.17 (1.10,1.29)	0.239**†**

Data format: Mean ± SD; *t*-test. †Median (interquartile range); Mann–Whitney U test. Significance: Bold *p-values indicate significant differences.

LPAs: Lipid-poor adenomas; PHEOs: Pheochromocytomas; CTu/CTa/CTv: Unenhanced/arterial/venous phase attenuation (HU); DEAP/DEVP: Arterial/venous enhancement (CTa − CTu, CTv − CTu); DEmax: Peak enhancement (max [DEAP, DEVP]); EPmax: Normalized enhancement (DEmax/CTu); LD/SD: Long/short diameter (mm); APW/RPW: Absolute/relative percentage washout; EP: Enhancement potential.

**Table 2:** Qualitative data characteristics between LPAs and PHEOs.

Characteristics	Washout group		*P*-value	Non-washout group		*P*-value	ALL subjects group		*P*-value
	LPAs (*n* = 31)	PHEOs (*n* = 29)		LPAs (*n* = 101)	PHEOs (*n* = 68)		LPAs (*n* = 132)	PHEOs (*n* = 97)	
Gender									
Men	7 (22.6)	17 (58.6)	**0.004**	48 (47.5)	35 (51.5)	0.615	55 (41.7)	52 (53.6)	0.074
Women	24 (77.4)	12 (41.4)		53 (52.5)	33 (48.5)		77 (58.3)	45 (46.4)	
Location									
Right left	14 (45.2) 17 (54.8)	14 (48.3) 15 (51.7)	0.809	50 (49.5) 51 (50.5)	29 (42.6) 39 (57.4)	0.381	64 (48.5) 68 (51.5)	43 (44.3) 54 (55.7)	0.533
Shape									
Round (0) Oval (1) Irregular (2)	12 (38.7) 17 (54.8) 2 (6.5)	10 (34.5) 15 (51.7) 4 (13.8)	0.631	38 (37.6) 55 (54.5) 8 (7.9)	26 (38.2) 26 (38.2) 16 (23.5)	**0.001**	50 (37.9) 72 (54.4) 10 (7.6)	36 (37.1) 41 (42.3) 20 (20.6)	**0.011**
Calcification									
Yes No	4 (12.9) 27 (87.1)	2 (6.9) 27 (93.1)	0.731	5 (5) 96 (95)	8 (11.8) 60 (88.2)	0.103	9 (6.8) 123 (93.2)	10 (10.3) 87 (89.7)	0.344
Cystic degeneration									
Yes No	2 (6.5) 29 (93.5)	17 (58.6) 12 (41.4)	**<0.001**	5 (5) 96 (95)	51 (75) 17 (25)	**<0.001**	7 (5.3) 125 (94.7)	68 (70.1) 29 (29.9)	**<0.001**
Haemorrhage									
Yes No	0 (−) 31 (100)	1 (3.4) 28 (96.6)	0.973	3 (3) 98 (97)	4 (5.9) 64 (94.1)	0.591	3 (2.3) 129 (97.7)	5 (5.2) 92 (94.8)	0.241
Intratumoural vessel									
Yes No	4 (12.9) 27 (87.1)	12 (41.4) 17 (58.6)	**0.013**	9 (8.9) 92 (91.1)	25 (36.8) 43 (63.2)	**<0.001**	13 (9.7) 119 (90.2)	37 (38.1) 60 (61.9)	**<0.001**
HBp									
Yes No	13 (41.9) 18 (58.1)	15 (51.7) 14 (48.3)	0.448	49 (48.5) 52 (51.5)	34 (50.0) 34 (50.0)	0.850	62 (47) 70 (53.0)	49 (50.5) 48 (49.5)	0.596
Laboratory index									
Normal abnormal	17 (54.8) 14 (45.2)	15 (51.7) 14 (48.3)	0.809	52 (51.5) 49 (48.5)	31 (45.6) 37 (54.1)	0.452	69 (52.3) 63 (47.7)	46 (47.4) 51 (52.6)	0.468
Symptom									
Yes no	7 (22.6) 24 (77.4)	7 (24.1) 22 (75.9)	0.887	18 (17.8) 83 (82.2)	20 (29.4) 48 (70.6)	0.077	25 (18.9) 107 (81.1)	27 (27.8) 70 (72.2)	0.112

Data are numbers of lesions. Data in parentheses are percentages. *P* values written in bold indicate a significant difference between lesions. PHEOs: Pheochromocytomas. LPAs: lipid-poor adenomas.

**Table 3:** The AUC and its 95% confidence interval (95% CI) with three different classifiers.

Group	Classifier	Sensitivity	Specificity	Accuracy	AUC (95% CI)
Washout group	Decision tree	0.999	0.955	0.978	0.977 (0.934–1.000)
	Support vector machine	0.917	0.818	0.870	0.926 (0.846–1.000)
	Logistic regression	0.958	0.727	0.848	0.915 (0.830–1.000)
No-washout group	Decision tree	0.999	0.966	0.985	0.983 (0.962–1.000)
	Support vector machine	0.987	0.847	0.927	0.961 (0.929–0.993)
	Logistic regression	0.999	0.814	0.920	0.968 (0.939–0.997)

In the washout group, the DT model achieved an AUC of 0.977 (95% CI: 0.934–0.999; accuracy 0.978), outperforming SVM (AUC 0.926, 95% CI: 0.846–0.999; accuracy 0.870) and LR (AUC 0.915, 95% CI: 0.830–0.999; accuracy 0.848). Similarly, in the non-washout group, DT attained an AUC of 0.983 (95% CI: 0.962–0.999; accuracy 0.985), compared to SVM (AUC 0.961, 95% CI: 0.929–0.993; accuracy 0.927) and LR (AUC 0.968, 95% CI: 0.939–0.997; accuracy 0.920).

Although the DT model demonstrated numerically superior AUC values, pairwise comparisons via the DeLong test indicated that these differences did not reach statistical significance among the three models in either the washout or non-washout groups (*p* > 0.05 for all comparisons, Table 4). The qualitative imaging features are detailed in Table 2. Cystic degeneration was significantly more prevalent in PHEOs than in LPAs across both cohorts (Washout: 58.6% vs. 6.5%, *p* < 0.001; Non-washout: 75.0% vs. 5.0%, *p* < 0.001). Similarly, the presence of intratumoural vessels was observed significantly more frequently in PHEOs than in LPAs (Washout: 41.4% vs. 12.9%, *p* = 0.013; Non-washout: 36.8% vs. 8.9%, *p* < 0.001).

**Table 4:** The delong test was performed on the within-group models of each group.

Group	Model classifier	Standard Error	Approximation		AUC Variance
			Z	*P*-value	
Washout group	Decision tree: support vector machine	0.838	0.0609	0.9515	0.051
	Decision tree: logistic regression	0.912	0.0680	0.9458	0.062
	Support vector machine: logistic regression	1.147	0.00958	0.9923	0.011
No-washout group	Decision tree: support vector machine	0.372	0.0591	0.9529	0.022
	Decision tree: logistic regression	0.347	0.0433	0.9655	0.015
	Support vector machine: logistic regression	0.432	−0.0162	0.9871	−0.007

### The DT interpretation and feature importance

**Figure 3:**
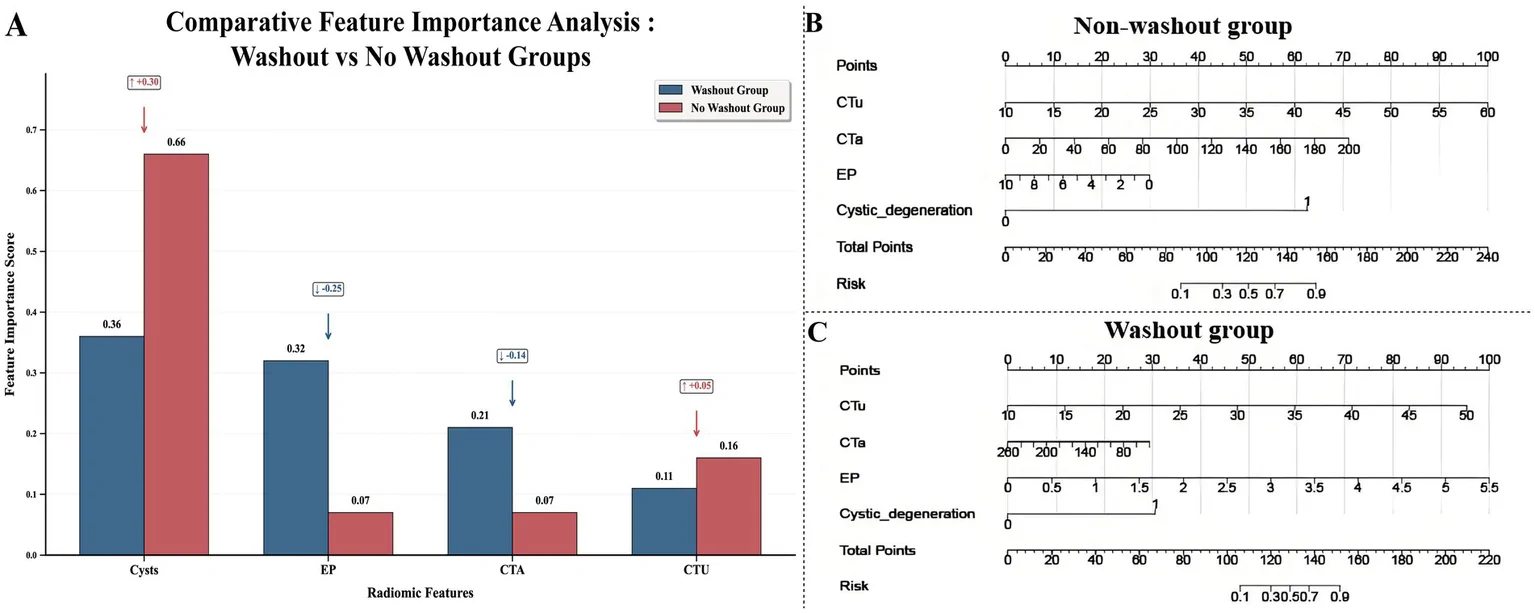
Feature importance comparison and cohort-specific risk scoring models. **(A)** Importance scores from the DT classifier for key CT-derived features in the washout (blue) and non-washout (red) groups. Arrows denote the magnitude and direction of between-group differences (e.g., ↑ + 0.30 for Cysts). **(B)** Point-based scoring system for the non-washout group, integrating CTu, CTa, EP, and cysts to generate a total score (0–240) linked to predicted risk. **(C)** Corresponding scoring system for the washout group, with CTu, CTa, EP, and cysts contributing to a total score (0–220) calibrated to risk probability.

To identify which imaging features contributed differently to classification in each subgroup, we compared feature importance scores between the washout and non-washout cohorts (Figure 3A). Cysts showed substantially greater discriminative weight in the non-washout group (importance = 0.66) than in the washout group (0.36). In contrast, EP and arterial-phase attenuation (CTa) were more influential in the washout group (EP: 0.32; CTa: 0.21) compared with the non-washout group (EP: 0.07; CTa: 0.07). Unenhanced attenuation (CTu) played a modestly larger role in the non-washout cohort (0.16 vs. 0.11).

Guided by these patterns, we constructed distinct clinical scoring systems for each group to translate model outputs into actionable risk estimates (Figures 3B,C). The non-washout scoring system incorporated CTu, CTa, EP, and cysts, yielding a total point range of 0–240 (Figure 3B). For the washout group, the system placed greater emphasis on CTa and EP, with a maximum score of 220 (Figure 3C). In both cases, cumulative points were mapped to predicted probabilities of PHEO, facilitating risk-stratified management.

**Figure 4:**
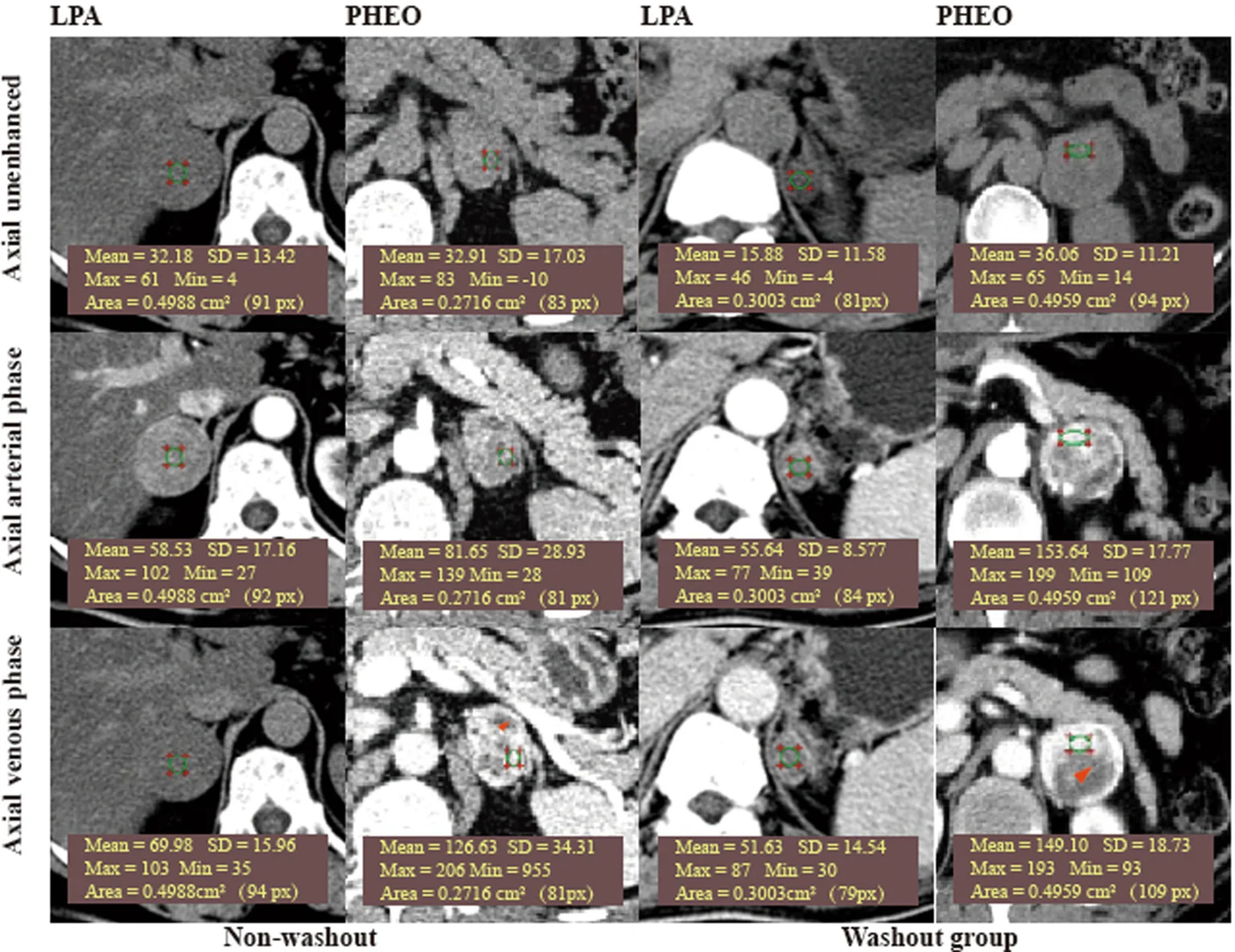
Representative CT images of LPAs and PHEOs in both cohorts. Non-washout group: (1) The patient with a right LPA was a 59-year-old female, and the CTu, CTa, and CTv values were 32.18, 58.53, and 69.98, respectively. The LD and SD of the mass were 39 and 35 mm. No evidence of cystic degeneration was observed within the tumour. (2) The left PHEO patient was a 55-year-old female with CTu, CTa, and CTv values of 32.91, 81.65, and 126.63, respectively. The LD and SD of the mass were 31 and 24 mm. The presence of cystic degeneration within the tumour was indicated by a red triangle. Washout group: (1) The patient with one LPA was a 57-year-old female, and the CTu, CTa, and CTv values were 15.18, 55.64, and 51.64, respectively. The LD and SD of the mass were 20 and 14 mm. No evidence of cystic degeneration was observed within the tumour. (2) The right PHEO patient was a 55-year-old female with CTu, CTa, and CTv values of 36.06, 153.64, and 149.10, respectively. The LD and SD of the mass were 37 and 31 mm. The presence of cystic degeneration within the tumour was indicated by a red triangle.

Representative clinical cases illustrating the application of these decision rules are presented in Figure 4.

**Figure 5:**
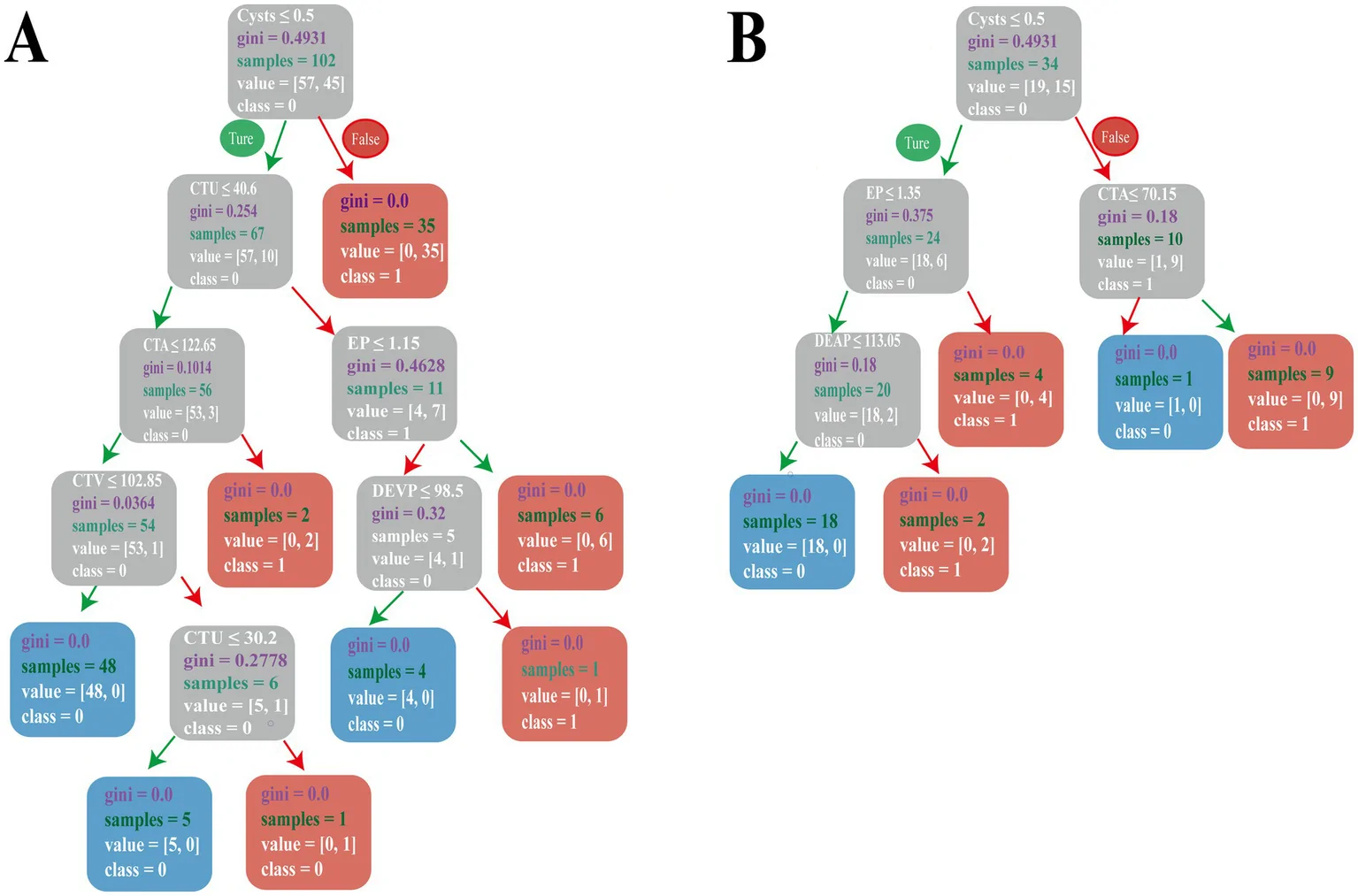
The DT structures visualizing the classification rules for the **(A)** non-washout and **(B)** washout cohorts. DT classification of the LPAs and PHEOs. The DT is examined using Classification and Regression Tree (CART) analysis. Decision nodes (square boxes) represent the CT features and splitting criteria used for classification. Cysts at the top are the root nodes. Results (0 = LPAs, 1 = PHEOs) are displayed in rounded angle boxes.

The corresponding decision rules extracted from the DT model structure are visualised in Figure 5. In the non-washout group (Figure 5A), the presence or absence of cystic degeneration served as the root node. Lesions without cystic degeneration were then stratified by CTu; a threshold of ≤18.8 HU strongly predicted LPA, while values >18.8 HU prompted further evaluation based on CTa. This hierarchical splitting effectively captures a clinically intuitive diagnostic pathway: first excluding the highly specific finding of cystic degeneration (favoring PHEO), then utilizing the well-established non-contrast attenuation threshold for adenomas. Using the entire non-washout cohort, cystic degeneration alone achieved a sensitivity of 75.0% (95% CI: 63.4–84.2%) and a specificity of 95.0% (95% CI: 89.1–98.1%) for diagnosing PHEO. In the washout group, the corresponding values were 58.6% (95% CI: 40.7–74.6%) and 93.5% (95% CI: 79.3–98.9%), respectively (see also Table 2). Beyond these root-node splits, the decision tree identified additional thresholds—such as CTu ≤ 40.6 HU and CTa ≤ 122.65 HU in the non-washout group, and EP > 1.35 and DEAP > 113.05 HU in the washout group—that further refined classification.

In the washout group (Figure 5B), the primary split was based on EP, followed by cystic degeneration and CTa. Lower Ep values were associated with LPAs, reflecting their relatively lower peak enhancement compared to the hypervascular PHEOs.

**Figure 6:**
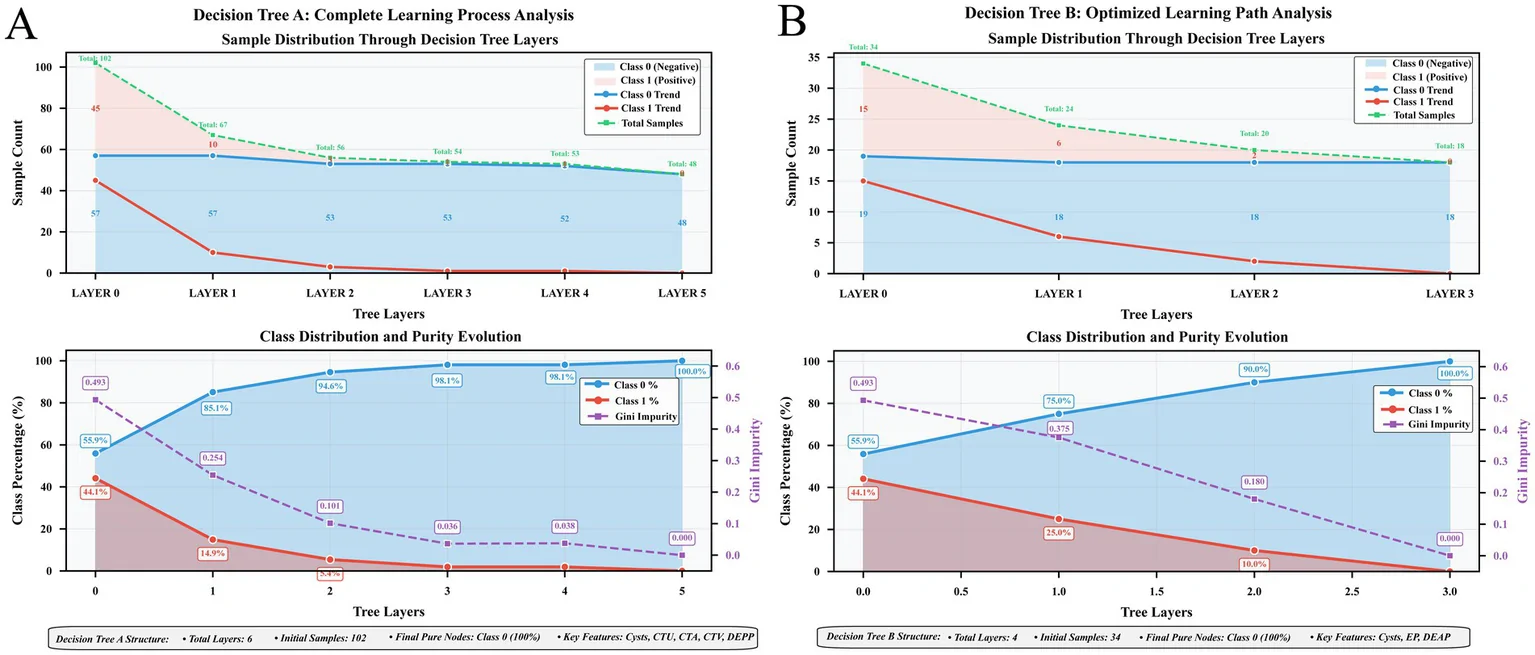
Analysis of DT learning processes. **(A)** Decision Tree A (complete learning process) charts the sample distribution and class parity evolution through each sequential layer, illustrating the progressive purification of classifications from root to terminal nodes. **(B)** Decision Tree B (optimised learning path) maps an efficient learning trajectory, highlighting the critical decision nodes that enable rapid and accurate differentiation between lipid-poor adenomas and pheochromocytomas. Charts within each layer represent the sample count (upper) and class percentage (lower) at the corresponding branch points.

The learning dynamics and sample stratification process of the DT models are further detailed in Figure 6. The DT A (Complete Learning Process) traces the evolution of class distribution and purity across all hierarchical layers, demonstrating the model’s step-wise refinement. In contrast, the DT B (optimised Learning Path) delineates a more efficient route to classification, rapidly isolating key discriminative features—primarily cystic degeneration and EP—to achieve diagnostic certainty with a concise branching structure. This visualization underscores the model’s capacity to emulate a logical, clinically coherent decision-making pathway.

## Discussion

This study demonstrates that an interpretable ML framework based on multi-phase CECT features can effectively differentiate PHEOs from LPAs. Our key finding is that the DT model demonstrated consistently high diagnostic performance across both washout and non-washout cohorts, achieving AUCs exceeding 0.97. Although the DT model demonstrated numerically superior AUC values, pairwise comparisons using the DeLong test indicated that these differences did not reach statistical significance. Consequently, the principal advantage of the DT model lies not in superior predictive accuracy, but in its unique ability to offer an optimal balance of high discriminatory power and inherent interpretability—a strength further amplified by the accompanying clinically accessible nomograms.

A critical question is whether this ML framework offers incremental value over conventional CT interpretation. While our DT model’s decision rules align with radiological reasoning—a feature that enhances its clinical trustworthiness—its strength lies in the objective, multi-parametric integration of features. Traditional approaches often rely on sequential, single-threshold rules (e.g., unenhanced CT ≤ 10 HU, or APW > 60%). In contrast, our ML models simultaneously weigh multiple features—including CTu, CTa, EP, and cystic degeneration—to identify optimal decision boundaries that maximise overall diagnostic accuracy. This multi-parametric approach provides a reproducible and mathematically grounded framework that complements, rather than replaces, conventional radiological assessment.

Direct comparison with conventional CT criteria provides quantitative context for our model’s performance. In a systematic review of adrenal washout CT, Woo et al. reported a pooled specificity of 67% for differentiating PHEO from adenoma, with 35% of PHEOs meeting adenoma washout criteria (Woo et al., 2018). Patel et al. similarly found that 33% of PHEOs met washout criteria on complete triphasic CT (Patel et al., 2013). In our cohort, the DT model achieved specificity >0.95 (Table 3). This difference—67% versus >95%—suggests that multi-parametric integration of CT features may improve diagnostic performance compared with single-threshold washout criteria alone.

Regarding prior ML studies, Mendi & Gülbay reported an AUC of 0.975 using a random forest model with 63 radiomic features on single-phase contrast-enhanced CT (Mendi and Gulbay, 2023), while Liu et al. achieved an AUC of 0.917 with LR on non-contrast CT features (Liu et al., 2022). Glazer et al. reported an F1 score of 0.96 using a rule-learning model with three radiomic features on portal venous phase CT (Glazer et al., 2025). Our DT model, by contrast, uses clinically familiar parameters (CTu, CTa, EP, and cystic degeneration) rather than abstract radiomic features, providing transparent decision rules (Figure 5) and nomograms (Figures 3B,C) that clinicians can interpret without specialised software.

The interpretability of the DT model yielded validated, data-driven insights into the relative importance of specific CT features, effectively demystifying the model’s decision-making process. The prominence of cystic degeneration as the dominant predictor, particularly in the non-washout cohort (Gini importance: 0.66), aligns perfectly with established literature and the known pathological hallmark of PHEOs (Liu et al., 2022; Niu et al., 2022; Motta-Ramirez et al., 2005). This finding is consistent with recent multi-centre studies and texture analysis research that have identified cystic changes as a key differentiator between PHEOs and adenomas (Shan et al., 2025; Altay et al., 2023; Phadte et al., 2024). While previous studies, including the radiomics analysis by Mendi & Gülbay, have achieved high accuracy using complex models like random forests (AUC 0.975) (Mendi and Gulbay, 2023), our work makes a distinct contribution by prioritising—and achieving—unparalleled transparency over mere predictive performance. The DT model directly addresses the “black-box” concern prevalent in high-dimensional radiomics, providing clear, hierarchical decision rules that explicitly highlight the pivotal role of cystic degeneration, EP, and baseline attenuation. This effectively translates a key pathological finding into a clinically intuitive and actionable metric, thereby fostering greater trust and facilitating adoption. We acknowledge that the specific CT criteria for cystic degeneration (CTu < 20 HU, DEAP < 10 HU, DEVP < 10 HU) were derived from a seminal study published in 1998 (Patel et al., 2013). However, these criteria remain widely referenced in subsequent radiomics and machine learning studies for adrenal tumour characterisation (Altay et al., 2023; Moawad et al., 2021), and the underlying principle—identifying non-enhancing, low-attenuation areas consistent with cystic or necrotic components—is grounded in robust pathophysiological reasoning. We therefore consider these criteria to remain a valid discriminator in the current study.

The integration of dynamic enhancement parameters was critical to our model’s success. The novel parameter, EP, which normalises the maximum enhancement to the baseline attenuation (CTu), helped mitigate confounding effects from intrinsic lesion density. Physiologically, EP reflects the maximal contrast uptake of the tumour during the early perfusion phases. The observation that EP differed between LPAs and PHEOs in a subgroup-specific manner suggests that the two tumour types exhibit distinct haemodynamic profiles, which may be attributable to differences in vascular architecture and perfusion characteristics. We observed an inverse correlation between CTu and EP: LPAs, with their characteristically lower baseline attenuation, exhibited higher relative enhancement ratios, whereas the inherently hyperdense PHEOs showed proportionally lower EP values despite strong absolute enhancement. This counter-intuitive finding may be explained by underlying pathophysiological differences: the more homogeneous vascular architecture of LPAs may facilitate more efficient and uniform contrast uptake relative to their baseline density. In contrast, the frequent presence of necrosis, cystic degeneration, and heterogeneous vascularity in PHEOs could diminish their normalised EP, even when absolute enhancement is high. This observation corroborates recent work highlighting the value of arterial phase enhancement patterns in distinguishing PHEOs, particularly in washout-indeterminate cases (Phadte et al., 2024). This synthesis of static and dynamic CT features provides a more physiologically grounded and nuanced differentiation method compared to approaches relying solely on non-contrast CT (Xiao et al., 2023; Yi et al., 2018).

Our multi-phase framework addresses a key limitation of conventional CT interpretation, where a substantial proportion of PHEOs may meet traditional CT washout criteria for adenomas (Patel et al., 2013). Recent meta-analyses and multi-centre studies have confirmed that approximately one-third of PHEOs exhibit washout characteristics overlapping with adenomas, underscoring the need for advanced differentiation methods (Shan et al., 2025; Phadte et al., 2024). By capturing the dynamic vascular physiology of these tumours, our approach provides a more comprehensive assessment that usefully complements standard washout calculations.

The continuous thresholds identified by the decision tree, such as CTu ≤ 40.6 HU and EP > 1.35, were automatically optimised during model training. These thresholds are model-derived and, as with any data-driven cut-offs, require validation in independent cohorts before they can be considered for use as standalone clinical decision criteria.

## Clinical implications

The developed nomograms (Figures 3B,C) offer a practical and immediate tool for clinical application. They provide a structured, evidence-based framework to triage patients with indeterminate adrenal nodules, effectively standardizing the interpretation of multi-phase CT findings. For instance, a radiologist encountering a non-washout lesion on a picture archiving and communication system workstation can swiftly input CTu, CTa, and assess for cystic degeneration to generate a risk score. This quantitative output can directly inform reporting language and management recommendations: a low score supports LPA and conservative follow-up, while a high score should trigger definitive biochemical testing and surgical referral for suspected PHEO. This has the potential to reduce diagnostic subjectivity and prevent both unnecessary interventions and missed diagnoses.

The transparency of the DT model’s decision rules is fundamental to enhancing clinical trust and facilitating its adoption. Our approach, which yields a simple, rule-based model, aligns with the growing emphasis on interpretable artificial intelligence in medical imaging, as demonstrated by other studies that have also employed interpretable rule-learning models to identify succinct, clinically transparent feature sets for adrenal tumour classification (Moawad et al., 2021). Unlike “black box” models, the clear hierarchical structure of the DT model allows clinicians to understand and verify the reasoning behind each prediction, ensuring the model’s decision-making process aligns seamlessly with clinical intuition.

## Limitations and future directions

This study has several limitations. Its retrospective nature may introduce selection bias. The most significant limitation is the single-centre design and the consequent lack of an external validation cohort. While we employed rigorous cross-validation techniques to mitigate overfitting, the generalisability of our model needs to be confirmed in future prospective, multi-centre studies. The relatively modest sample size, particularly within the washout group (n = 60), also underscores the need for validation in larger, more diverse populations. We are planning external validation using independent multi-centre cohorts, applying the developed DT model and the derived nomograms to prospective data from collaborating institutions while evaluating both diagnostic performance and inter-reader reproducibility in real-world clinical settings.

Furthermore, the absence of systematic biochemical data—such as plasma-free metanephrines—for the entire cohort represents a significant constraint. Without these data, we were unable to correlate imaging phenotypes with hormonal activity or definitively exclude the possibility of biochemically silent PHEOs or, conversely, functioning adenomas within our cohorts. Consequently, our model is strictly limited to differentiating tumour types based on imaging characteristics and should be regarded as a complementary tool within a comprehensive diagnostic workflow that necessarily includes biochemical evaluation. We also acknowledge the absence of delayed-phase CT data. Delayed imaging is not part of many routine abdominal CT protocols.

Additionally, the feature set was predefined according to established clinical knowledge and literature. While this approach ensures clinical relevance and interpretability, it may overlook certain discriminative imaging characteristics that are not yet well documented. Future efforts should prioritize external validation across multi-centre cohorts to rigorously assess generalizability (Xie et al., 2025), exploration of a wider spectrum of radiomic features to better capture tumour heterogeneity (Altay et al., 2023; Zhou et al., 2025; Zhao et al., 2024; Liu et al., 2024), and the development of hybrid models that integrate both imaging and biochemical data for a more holistic diagnostic strategy.

Despite the absence of delayed-phase data, our model achieved high diagnostic accuracy. This finding suggests that the additional radiation dose and scan time associated with delayed acquisition may not be necessary when using this ML-based framework—particularly for adrenal lesions incidentally discovered on routine CT where a dedicated adrenal protocol is not immediately available.

## Conclusion

Accurately distinguishing between LPAs and PHEOs is paramount for directing appropriate clinical management. Our study demonstrates that an interpretable ML framework based on multi-phase CECT features can achieve high diagnostic accuracy. The DT model, in particular, offers an optimal combination of performance and interpretability. By synthesising dynamic enhancement patterns with baseline morphological and attenuation characteristics, and by providing transparent decision rules that align with clinical reasoning, this approach addresses key limitations of conventional CT interpretation and provides a clinically actionable and trustworthy tool for distinguishing these challenging adrenal tumours.

## Data Availability

The original contributions presented in the study are included in the article/supplementary material, further inquiries can be directed to the corresponding author.

## Glossary

AI: Adrenal incidentaloma
APW: Absolute percentage washout
AUC: Area under the curve
CECT: Contrast-Enhanced Computed Tomography
CI: Confidence Interval
CT: Computed Tomography
CTa: Arterial Phase CT Attenuation
CTu: Unenhanced CT Attenuation
CTv: Venous Phase CT Attenuation
DEAP: Degree of Enhancement in Arterial Phase
DEVP: Degree of Enhancement in Portal Venous Phase
DT: Decision Tree
EP: Enhancement Potential
HU: Hounsfield Units
LD: Long Diameter
LPA: Lipid-Poor Adenoma
LR: Logistic Regression
ML: Machine Learning
PHEO: Pheochromocytoma
ROI: Region of Interest
ROC: Receiver Operating Characteristic
RPW: Relative Percentage Washout
SD: Short Diameter
SVM: Support Vector Machine
